# The everlasting hunt for new ice phases

**DOI:** 10.1038/s41467-021-23403-6

**Published:** 2021-05-26

**Authors:** Thomas C. Hansen

**Affiliations:** Institut Max von Laue-Paul Langevin, Grenoble Cedex, France

**Keywords:** Structure of solids and liquids, Structure of solids and liquids

## Abstract

Water ice exists in hugely different environments, artificially or naturally occurring ones across the universe. The phase diagram of crystalline phases of ice is still under construction: a high-pressure phase, ice XIX, has just been reported but its structure remains ambiguous.

Water is the molecule that makes our planet so special and gives it its nickname, the blue planet. Liquid water is the medium for any form of life on Earth and is considered to be a *sine qua non*-condition for life on any place in our universe. Ice, the solid phase of water, has always been in the focus of science. It was maybe even ice, that was a necessary substrate for first life on Earth to emerge^1^; Therefore, it is normal to be very curious about all possible structures of water ices under different conditions—twenty crystalline and further amorphous forms are now known to exist (Fig. 1).

**Fig. 1:**
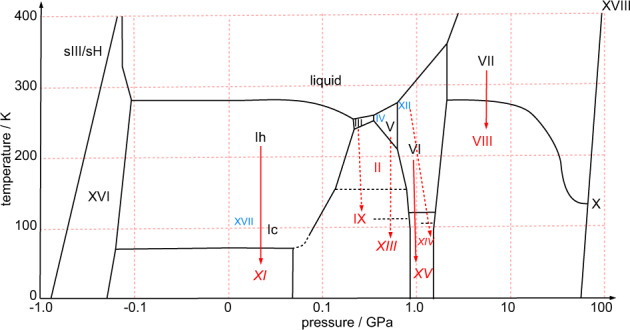
Schematic phase diagram of crystalline ice phases inspired by Bartels-Rausch et al.^1^, Salzmann et al.^18^ and Huang et al.^21^. Phases labeled with black Roman numerals are hydrogen-disordered phases; those labeled with red numerals are hydrogen-ordered phases; those labeled with blue numerals are metastable phases. The red solid arrows represent direct disorder-order transitions. The red dotted arrows represent disorder-order transitions that cross the stability range of another phase.

Ice I_h_, hexagonal ice, is the only form relevant to the Earth’s environmental conditions, as even the thickest ice slabs do not provide the necessary pressure to pass it into high-pressure polymorphs. However, so-called cubic ice, ice I_c_,^2^ only very recently isolated in pure form^3,4^, plays a role at very low temperatures in the higher atmosphere in the nucleation of ice^5^; ice VI, the lowest high-pressure ice phase at room temperature has been spectroscopically found in diamond inclusions^6^, and ice VII has been speculated to be present on Earth in cold subduction zones^7^. In this context we should remember that the word ‘crystal’ originates from the antique Greek word for ice, *κρύος*: the ancient Greeks thought that crystals of quartz — as found in the mountains as ‘rock crystal’ — were just ice crystals which had been cooled so severely in the cold mountain climate, that they became solidified forever.

In this issue of Nature Communications, we see a third paper on a newly discovered ice XIX,^8^ after the first two reports on this phase appeared, only few weeks ago in this journal^9,10^. The structural results are not unambiguous, though, as we will see.

With the most recent publications on an ice XIX in this journal, twenty crystalline phases of water ice are now identified. Most of these phases occur at high pressure; however, two of the most recently discovered phases of ice^11–13^ are negative pressure ones – they result from emptying the cages of gas hydrates and show an extraordinarily low density. For most of the high-pressure phases, there is a correspondence between a hydrogen-disordered phase at high temperature and a hydrogen-ordered one at lower temperature. Hydrogen ordering appears spontaneously upon cooling, but some hydrogen-disordered phases need the help of a dopant to overcome a kinetic hindering, otherwise they remain metastable at low temperatures. It was obviously discussed whether a hydrogen-ordered phase after doping with, for instance, an acid, can still be called a ‘valid’ structure of water ice. Yet, since the discovery of ice XI in 1972^14^ and its structure determination in 1997^15^, the approach has been eventually widely accepted and has been applied in the discovery of the ices XIII and XIV^16^, XV^17^ — and now XIX.

The ordering from ice I_h_ to XI, from V to XIII, from XII to XIV needs to be induced through basic or acidic doping, as the ordering temperatures are too low to observe spontaneous ordering in laboratory time scales. However, in the case of ordering of ice VI to XV a more complex kinetics has been observed. The ordering temperature tails from a relatively high 129 K down to 100 K, and the process depends on the applied pressure. Also, the experimentally found (partial) ordering of hydrogen differs from what is predicted by computer simulation^18^.

Back in 2018, Gasser et al. reported on a ‘β-ice XV’ phase^19^, obtained, like the hydrogen ordered phase ice XV, upon cooling ice VI. At that time, experimental evidence was insufficient to attribute a Roman numeral to this phase – as is the convention for crystalline phases of ice – because its structure remained to be elucidated.

Now, in a recent paper, Yamane et al.^9^ investigate this high-pressure phase and describe it as a hydrogen-ordered phase of ice VI, with a different hydrogen ordering as compared to ice XV, and assign to it a new Roman numeral, XIX. While they can show convincingly that ice XIX is different from ice XV and ice VI, by means of dielectric measurements and in situ neutron powder diffraction, its crystal structure remains ambiguous: several space groups remain possible according to the authors’ data on the deuterated in situ high-pressure samples of ice XIX… Ice XIX definitely forms a $\sqrt{2}\times \sqrt{2}\times 1$ super-cell with twice the unit cell volume of ice VI (and ice XV). Neither orthorhombic nor tetragonal unit cells can be ruled out convincingly against each other. As we will learn later, it is not even clear whether it is the hydrogen ordering, which is different compared to ice VI and ice XV, although it remains the working hypothesis — and the selling argument, or headline, of their manuscript. The dielectric measurements indicate an ordering of hydrogen as compared to ice VI, but whether the topology of this order is different from ice XV, as the title suggests, may be contested.

At the same time, Gasser et al. have investigated the same phase of ice, and published a paper on the structure of ice XIX^10^, again announced as a second (different from ice XV) hydrogen-ordered polymorph to ice VI. They performed ex situ high resolution neutron powder diffraction on recovered samples, providing better quality data as compared to in situ high-pressure data. The work also sheds more light on the phase boundaries, such as the order-order transition between ice XV and ice XIX. Yet, by a crystallographically different approach (filtering of eligible subgroups by compatibility with the oxygen lattice), an ambiguity between different possible space groups remains. To reduce the 109 possible solutions in five space groups to a smaller set of likely solutions, they referred to the preprinted paper of Yamane et al. and limited the quantitative tests to Yamane’s structure models in the three space groups both groups found to be eligible. Again, even a distinction of orthorhombic and tetragonal solutions remains ambiguous. The oxygen topology is found to be the same in ice VI, XV and XIX. The hydrogen order is only partial in both, ice XV and XIX. And in both cases, the ordering is found to be anti-ferroelectric (in contrast to initial suspicions to find a ferroelectric ordering like in ice XI). Two space groups are most likely, tetragonal *P*$\overline{4}$ or orthorhombic *Pcc2*. It would be easy to distinguish the solutions as *Pcc2* should exhibit pyroelectricity and non-polar *P*$\overline{4}$ piezoelectricity, but for this one would need single crystals which are very likely inaccessible.

It is not surprising that the phase, which had been called β-ice XV previously, which is found different from ice XV and therefore called ice XIX, is suspected to show hydrogen ordering, and a different one as compared to ice XV. How H atoms order and whether an ordered structure is ferroelectric or anti-ferroelectric remains difficult to predict, a fact which nourished the suspicion that not only one H-ordering could be possible. When, in the ordering of ice VI to ice XV, calorimetry suggested a second, underlying, process, and that both ordering processes would have entropy changes corresponding to only partial H-ordering, a different hydrogen ordering became the likely candidate. Yet, this expectation biases the scientist’s look at experimental results and may limit the possible interpretations of the experiment.

In addition, as for the formation of other hydrogen-ordered phases, certain doping strategies were needed to obtain the new ice phase. And here we eventually run in some danger, as every group has its own recipe to obtain ice XIX, can we really be sure to look at the same phase in all three papers?

The third paper on ice XIX^8^, published in Nature Communications only a few weeks later than the other two, comes from a team, Salzmann et al.^16,17^, which has quite some experience in the hunt for hydrogen-ordered ice phases. The structures of ice XIII, XIV and XV have already been determined as well by Salzmann et al. As in the previous investigations, upon cooling a doped sample at higher pressure, additional Bragg peaks appear which imply an increase of the ice VI unit cell to a $\sqrt{2}\times \sqrt{2}\times 1$ super-cell. However, the authors consider local distortions of the two individual networks of the ‘self-clathrate’ structure of ice VI. They consider all possible permutations of — respectively — tilting, shearing or’squishing’ the hexameric clusters of water molecules, the characteristic building unit in the ice VI structure which leads to — respectively — three, two or again three different space groups, all subgroups of the space group symmetry of ice VI and compatible with the super-cell. Only one space group, *Pbcn*, allows for the observed additional Bragg peaks (and all forms of distortion). The space group allows a good fit of the diffraction data — together with continued total hydrogen disorder in contradiction to the two previous papers. The lower symmetry with respect to the one of ice VI justifies the assignment of the Roman numeral ‘XIX’ to this phase, but concerning the hydrogen ordering, ice XIX remains a deep glassy state of ice VI with pressure-induced distortions as already suspected earlier by Rosy-Finsen et al.^20^.

Although some weak hydrogen ordering cannot be excluded, it is not the main structural feature distinguishing it from ice VI (and ice XV). However, slightly different doping strategies have been applied, are we really looking at the same ice phase in all cases? Are the conclusions of Salzmann et al. compatible with Gasser et al.’s observations on the ice XIX to ice XV ‘order-order’ phase transition?

Surely, we have not seen the last paper on this new crystalline polymorph of ice yet. There is still additional proof to provide to support the solution found by the paper hereafter. If the last solution offered, the deep glassy state, is the right one, one may wonder whether there is a hydrogen-ordered phase of this distorted phase. Also, might a second hydrogen-ordered phase, as it was claimed by Yamane et al. and Gasser et al. a few weeks ago, exist for any hydrogen-ordered phase?

A remarkable common feature of all three papers is that they are based on neutron powder diffraction (at different facilities — J-PARC and ISIS — with different instruments and at different resolutions — HRPD and PEARL — with different experimental approaches — ex and in situ). I may conclude with this biased observation, the role of neutron diffraction in the investigation of ice structures remains crucial.
